# Editorial Extracts and Gleanings

**Published:** 1874-02

**Authors:** R. C. Word

**Affiliations:** Georgia


### EXTRACTS AND GLEANINGS.

BY R. C. WORD, M.D., OF GEORGIA.

*Hemorrhage in Abortion.*—One of the most embarrassing difficulties encountered by the obstetric practitioner is the profuse and often alarming hemorrhage in cases of abortion, resulting from a partially detached ovum or placenta. In the early stages of impregnation, when the ovum is partially detached and grasped by the cervex uteri, it is exceedingly small and difficult to reach. In this condition, the hemorrhage will continue. The tampon may suspend the hemorrhage, but cannot be relied upon as a permanent measure of relief. And the same is true of a retained placenta after the expulsion of the embryo. The placenta or the ovum, as the case may be, must be removed.

Dr. Meigs remarks, that "In some cases of abortion it is considered impracticable to remove the placenta. Attempts by ergotism, with a proper small forceps and with a placenta hook, should be carried as far as a sound discretion will permit; but all rude and forcible attempts to procure extraction should be regarded as equally dangerous as the continued stay of the afterbirth in the cavity, etc." Dr. Dewees and other writers advance similar views; and recently it has been proposed to use the placenta hook through a speculum as a safer method of handling the instrument.

Now, our object in penning this article is to give to the profession an opinion, founded upon experience, that *it is practicable in every case* to deliver the ovum or placenta, and that, too, without the dangerous, and as we believe, the impracticable forceps or placenta hook. This fact we learned as far back as the year 1836. Being called to a case in the country wherein the patient was flooding profusely from a partially detached ovum, at about the second month, and not being able to more than touch the os in the ordinary way, and finding that the woman must inevitably and speedily succumb if not promptly relieved, we *determined to deliver it.* Administering first a full dose of ergot, we raised the patient to an angle of about 45°, with her knees drawn up to the buttox, and forcibly and somewhat violently pressing the fundus of the womb with the left hand above the symphisis pubis, *we brought the womb within easy reach,* and quickly scooped out the ovum with the finger of the right hand. The woman—in a fainting condition—was at once let down, with the head below a level, and with stimulants, etc., soon reacted and had a good recovery.

There possibly might occur cases in which this plan could not be carried out; but this much we can truthfully say: that, with perhaps our full proportion of these cases, we have never failed to accomplish the desired result in every instance since the period above mentioned.

*Impassible Stricture.*—W. F. Trevan, Surgeon to the West London Hospital, remarks, in the *London Lancet*, that in cases of impassable stricture of the urethra, all efforts to pass the catheter having utterly failed, there are three operations open to us: First, the puncture of the bladder through the rectum; next, Syme's operation for impermeable urethra; and lastly, the old French operation called "*La boutonnière.*" He greatly prefers the last named operation, as it not only relieves the bladder immediately, but ultimately cures the stricture. He thus describes it:

"It is not a dangerous operation, and is not so difficult of execution as is supposed; for the portion of the urethra behind the stricture is always greatly distended, so that a dissection through the perineum for a depth of one inch and a half will nearly always be sufficient. Formerly I used metal instruments with which to perform this operation; now, however, I have simplified matters, and employ only a knife and a large olivary elastic catheter. My first object is to get into the bladder from the perineum. Having made my incision down the penile urethra, I pass the catheter into the wound, and with great gentleness endeavor to insinuate it into the membranous urethra. I have never failed to effect my object. Afterwards I pass the same catheter as far as it will go, and then by cutting on its point I liberate it and pass it into the wound, from whence I conduct it along the tip of my left forefinger into the bladder. I now never leave a catheter in the bladder, but content myself with passing it every other day at first, and twice a week after the first month. At the expiration of two months it will suffice to introduce the catheter once a week, and at a later period still less frequently. For some time after the operation all the urine comes by the wound, but it usually heals up without any trouble. The great advantages which this operation possesses over all others is, that it attacks the disease at its seat and does not injure any healthy part; it gives a free vent for all abscesses, and, by restoring the urethral canal, it finally cures both stricture and fistulæ. In the whole range of surgery there exists not an operation which can render such signal services to the sufferer.

Shingles.—In our own treatment of "shingles," we have found nothing so valuable to relieve the pain as the hypodermic use of morphia and atropine, while the eruption upon the face and near the eye is painted with tincture of iodine; so that if it is confounded with erysipelas—which it is apt to be—no harm can result to the patient. Nitrate of silver has been found to add to the patient's pain and discomfort, while it is proper to use a solution of atropine to relieve irritation of the nerves.—*Med. & Surgical Reporter.*

*The Bavarian Dressing for Fractures.*—The "Bavarian" dressing was first brought to the notice of English and American surgeons by Mr. Moffitt, of the English army, in the "appendix to the Army Medical Report for 1869." This report I have not seen, but in the Dublin Quarterly Journal for August, 1871, is a paper by Dr. Corley, of the Jervis St. Hospital, Dublin, in which a description of the dressing is given. This paper is republished in Braithwaite's Retrospect for January, 1872. Dr. Corley thus describes the dressing: "Two pieces of flannel, suited to the length of the limb, are cut sufficiently wide to overlap slightly in front. When so prepared they resemble the leg of a stocking cut vertically. One is now laid over the other, and they are stitched together from top to bottom down the mesial line, like two sheets of note paper stitched at the fold. They must now be spread out under the injured limb, so that the line of stitching corresponds to the back of the calf. The two inner leaves, so to speak, are now brought together over the shin and fastened by long pins, the heads of which are bent. The leg being held firmly, an assistant mixes the plaster with about an equal bulk of water, and rapidly applies it, partly with a spoon and partly pouring over the outer surface of flannel *covering the limb*. The two portions of the second layer are then quickly brought over, so as to meet, and the inequalities in the distribution of the plaster are removed before it hardens, by smoothing with the hand. In about three minutes the gypsum sets, and the limb is encased in a strong, rigid covering, which gives uniform pressure and support to every part. The edges of the flannel in front can now be trimmed, and the pins withdrawn from the inner layer by seizing their bent heads. A couple of strips or a few turns of the roller make all secure. In order to take the apparatus off, it is only necessary to remove the strips and separate the edges of the flannel, when the two sides fall asunder, the line of stitching behind acting as a hinge." The only difficulty that I have found in the application of this dressing is in the removal of the pins fastening the inner layer of flannel. In three cases, more than half of the time occupied was spent in finding and removing these pins after the plaster had hardened. I am in hopes that some experiments that I am now occupied with will result in my finding a readily removable substitute for the pins.

It is not alone to fractures that this dressing is applicable. It is equally as well adapted to acute and chronic inflammations of joints, where, as every one knows, perfect rest of the articulation is of the greatest value.

*Eczema Genitale.*—Dr. J. M. Hyde, of Chicago, reports six cases of obstinate Eczema occurring about the nates and genitals which were relieved by the use of an ointment of belladonna and sulpho-carbonate of zinc. The strength of the ointment should vary with the degree of violence of the symptoms. The following may be used as an average: ℞ Ext. belladonna, gr. xx.; lard, ℥i; sulpho-carb. zinc, gr. x. M.

*Fatal Suffocation from Nitrous Oxide Gas.*—The first death in England from the inhalation of nitrous oxide gas, is reported in the *Lancet* for May, 1873. It was in the case of a lady for the extraction of a tooth. The gas having been safely used in previous cases, was undoubtedly pure.

It is stated that at the beginning of the administration, the lady's pulse became rapid and less full. At the proper degree of insensibility the inhalation was stopped, and the tooth extracted. Immediately after the operation the face became livid, and the features "commenced to swell." A short time was lost in procuring medical assistance, and a very few minutes thereafter her pulse ceased to beat.

No *post mortem* was had in this case, but no doubt is expressed that the cause of death was "paralysis of respiration." There was no obstruction to the air passages, but she was as powerless to breathe as though she had been immersed in water. The opinion is avowed that nitrous oxide is not an anæsthetic in the proper sense of the word, but acts by suspending, for a brief period, the organic process of respiration. The insensibility produced by the gas is afforded during an interval of partial death. Generally, the suspended function returns, but not always, as this case proves.

*Veratrum in Inflammation.*—As a rule, the remedies that will cure fever will cure inflammation. To this I believe there are no exceptions, if a proper diagnosis is made, and we are governed by the same indications for prescribing. Aconite, veratrum, gelseminum, belladonna, nux vomica, quinia, and other direct remedies, may be prescribed with the same certainty in inflammation as in fever. There is the same necessity for securing a good condition of stomach and upper intestine for digestion, and giving proper food. The same necessity for securing normal waste and excretion, and having the tissues renewed from good blood. The pallid tongue calls for alkalies, the dark redness of mucous membranes for acids, the pasty white coat for the sulphites, etc.

My experience teaches me that local inflammations are reached directly by this direct medication, and with a certainty a hundred times greater than by the old routine of internal and external counter-irritation. It makes no difference where its location, how great or how little, the treatment is exactly the same as for a fever presenting the same symptoms or indications for remedies.

It must not be expected that the indications for remedies will be as pronounced in the case of inflammation as in fever, but they are always sufficient.

I have treated inflammation of the lungs with veratrum alone, veratrum with gelseminum, veratrum with ipecac, epicac alone, aconite alone, with a success I never saw obtained from the use of nauseants and counter-irritation. Other cases required the use of the sulphites, of quinia, or the mineral acids. I am not alone in this experience, for scores of our more recent students, who have learned this practice in lectures, give testimony to its success.

The local application of veratrum, in the early stages of a superficial inflammation, will not unfrequently arrest its progress. In this way we use it in erysipelas, in phlegmonous inflammation of cellular tissue, in felons, diseases of the bones, tonsilitis, etc.—*By Dr. Scudder, in the Eclectic Med. Jour.*

*Sarracenia Purpura.*—This remedy was little known as one of great value until the last epidemic of small pox that appeared in the United States. The liberal members of the medical profession found this remedy much more than a diaphoretic and diuretic. They, by its exhibition in several thousand cases, have demonstrated the remedy to be not only a prophylactic, but a true curative agent in small pox. It seems to have some direct action upon the virus of the disease, altering and destroying its character. It has long been known that the sulphites or hyposulphites have a marked effect in checking the disease; but to the free use of a simple decoction of this drug, thousands of cases have been prevented, and a still larger number cured.

Warmth, stimulation, bathing with stimulating washes, nutritious diet.

Perhaps its chemical properties may have something to do with it, containing a large per centage of phosphorates and chlorides, and other agents of an alkaline character.

The thing is certain, that where the pitcher plant is drank freely, there will be no small pox; where it is given freely, and heat, bathing and nutritious diet in a bad case, it will either partially abort, or render it an extremely mild attack.—*Eclectic Med. Jour.*

*Taraxacin.*—Leontodon is a valuable remedy in all diseases of the digestive and hepatic systems. Its action is chologogue and tonic. The following is an excellent formula for its administration. ℞ Leontodon; hydrastin aa, grs. xxx; ext. nux vomica, grs. viij. M. Ft. pills No. xx. One thrice daily. Valuable in dyspepsia and constipation. Dose of the remedy, two to three grains.—*Eclectic Med. Journal.*

Dr. H. S. Reynolds, of Baltimore, says, through the *Eclectic Medical Journal*, that a grain or two of the acetate of lead, put into the cavity of a tooth, relieves a larger proportion of cases than any other known remedy.

---

