# Correction

**Published:** 1874-03

**Authors:** 


*Correction.*—In the article on *Hemorrhage in Abortion* in last issue of this Journal, the types make us say that the operation thus described was performed as far back as 1836, it should read 1856.

